# The Rigor and Transparency Index Quality Metric for Assessing Biological and Medical Science Methods

**DOI:** 10.1016/j.isci.2020.101698

**Published:** 2020-10-20

**Authors:** Joe Menke, Martijn Roelandse, Burak Ozyurt, Maryann Martone, Anita Bandrowski

**Affiliations:** 1 Center for Research in Biological Systems, UCSD, SciCrunch Inc, La Jolla, CA 92093, USA; 2 Independent Consultant at Martijnroelandse.dev, Amsterdam, the Netherlands; 3 Department of Neuroscience, UCSD, La Jolla, CA 92093, USA; 4 Department of Neuroscience, UCSD, SciCrunch Inc, La Jolla, CA 92093, USA

**Keywords:** Biological Sciences, Bioinformatics, Biological Sciences Research Methodologies, Methodology in Biological Sciences

## Abstract

The reproducibility crisis is a multifaceted problem involving ingrained practices within the scientific community. Fortunately, some causes are addressed by the author's adherence to rigor and reproducibility criteria, implemented via checklists at various journals. We developed an automated tool (SciScore) that evaluates research articles based on their adherence to key rigor criteria, including NIH criteria and RRIDs, at an unprecedented scale. We show that despite steady improvements, less than half of the scoring criteria, such as blinding or power analysis, are routinely addressed by authors; digging deeper, we examined the influence of specific checklists on average scores. The average score for a journal in a given year was named the Rigor and Transparency Index (RTI), a new journal quality metric. We compared the RTI with the Journal Impact Factor and found there was no correlation. The RTI can potentially serve as a proxy for methodological quality.

## Highlights

• Evaluated 1,578,964 articles for rigor criteria using SciScore, an automated tool
• Developed a journal quality metric and studied rigor criteria reporting trends
• No correlation between the Rigor and Transparency Index and the Impact Factor
• The Rigor and Transparency Index may serve as a proxy for methodological quality

## Graphical Abstract

**Figure:**
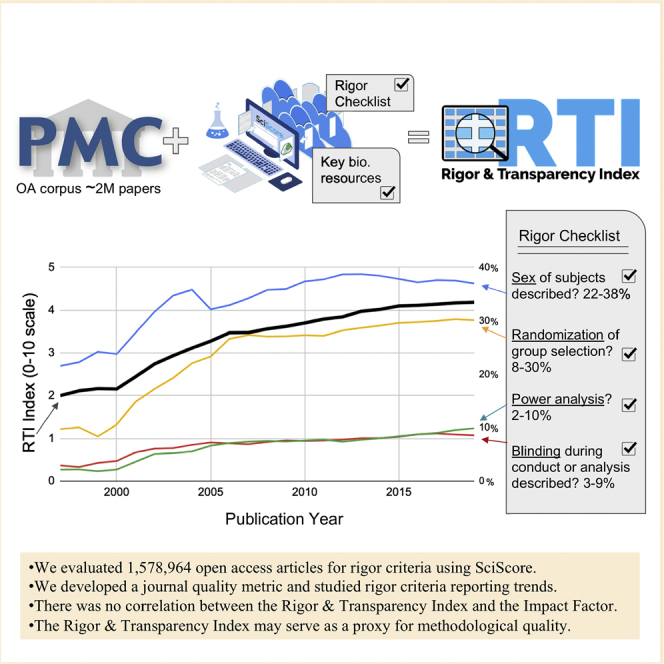

## Introduction

The National Institutes of Health (NIH) have designed and adopted a set of rigor and reproducibility guidelines expected to be addressed in grant proposals submitted to the NIH that cover the aspects of study design most likely to impact a study's reproducibility (for NIH Guidelines see NOT-OD-15-103 [National Institutes of Health, 2015]; see also EU Report Open Science Monitoring [RAND Europe, 2017]); for their intellectual underpinning [Landis et al., 2012]; for examples, Hackam and Redelmeier, 2006; van der Worp et al., 2010. Multiple journals have adopted similar guidelines in their instructions to authors [e.g., Nature Checklist (Nature Team, 2013)]).

The NIH guidelines, which list the most common components of other guidelines, are part of a growing list of recommendations and requirements designed to address different aspects of rigor and reproducibility in the scientific literature, e.g., the ARRIVE (Kilkenny et al., 2010), CONSORT (Schulz et al., 2010), STAR (Marcus, 2016), and RRID (Bandrowski et al., 2015) standards (for a full list of applicable standards please see the EQUATOR Network, https://www.equator-network.org/). The Animal Research: Reporting of *In Vivo* Experiments (ARRIVE) guidelines are a highly comprehensive and universally accepted set of criteria that should be addressed in every animal-based experiment. The guideline contains 39 items (20 primary questions and 19 subquestions). The Consolidated Standards of Reporting Trials (CONSORT) statement consists of a 25-item checklist along with a flow diagram governing how clinical trials should be reported. STAR methods (structured transparent accessible reporting) is a reporting framework developed by Cell Press aimed at improving reproducibility through, among other things, a standardized key resources table. The RRID Initiative, another reproducibility improvement strategy, asks authors to add persistent unique identifiers called research resource identifiers (RRIDs) to disambiguate specific assets used during experimentation. RRIDs can be considered as universal product codes (UPC) that identify the ingredients needed for an experiment. The initiative covers a wide variety of resources, including (but not limited to) antibodies, plasmids, cell lines, model organisms, and software tools. The initiative was started because antibodies were notoriously difficult to identify unambiguously in the published literature (Vasilevsky et al., 2013).

Unfortunately, studies of publishing practices generally find poor compliance by authors and enforcement by reviewers, even with the availability of checklists and instructions to authors; although, some journals do not even mention these guidelines to authors at all (Hirst and Altman, 2012). And even when authors assert that they follow ARRIVE, the evidence still shows that the guidelines are not followed (Hair et al., 2019; Kilkenny et al., 2009; Leung et al., 2018). This is not to say that authors and journals are the sole source of the problem as many research stakeholders contribute, including institutions who could improve guideline adherence as well as overall research quality by more effectively leveraging their influence on researchers (Moher et al., 2020; Rice et al., 2020). For example, *Nature*, one of the most prestigious and influential journals, through the work of the NPQIP collaborative group (Nature Publication Quality Improvement Project) verified that asking authors to use the Nature Checklist produced an improvement in rigor criteria (NPQIP collaborative group, 2020). In the case of RRIDs, the guidelines were not routinely followed when authors were asked by journals through instructions to authors or by checklists, but a direct request from the editors for RRIDs during the publication process proved highly effective in improving author compliance (Bandrowski et al., 2015). It might be an overgeneralization to say that authors do not follow guidelines unless they are prompted to do so; however, based on our anecdotal evidence, author compliance with guidelines is generally lower if there is no mechanism to verify compliance with the guideline.

In order to reduce confusion around the proliferation of guidelines and to improve author compliance, the above guidelines were incorporated into the Materials Design, Analysis, and Reporting (MDAR) framework, a recently released pan-publisher initiative enacted to create a consistent, minimum reporting standard that spans across all life sciences (Chambers et al., 2019). The MDAR checklist includes many of the elements that are present in the NIH, ARRIVE, CONSORT, and RRID standards. The checklist is not intended to supplant more granular reporting of information but rather is to be used as a generalist instrument across the biological research community to ensure that the minimum required reporting is met. However, if we are correct, on its own, the MDAR checklist will likely also have spotty author compliance unless it is enforced, a problem that could significantly benefit from automated text mining (Kilicoglu, 2017).

Studies that seek to investigate the degree to which authors comply with various guidelines have been limited to manual review of a few criteria because the task is highly labor intensive (Schulz et al., 1995; Sena et al., 2007). Prior work has explored the use of NLP techniques to automate clinical trial characteristic extraction and assessment (Kiritchenko et al., 2010; Marshall et al., 2015). Here we broaden and continue that work by introducing SciScore, an automated tool using natural language processing (NLP) and machine learning, which can be used by journals and authors to aid in compliance with the NIH rigor and RRID guidelines. The tool currently evaluates compliance with six key recommendations, largely shared between most guidelines and listed in Table 1, and checks for key resource identifiability for a variety of resource types. Here we show how this tool can be used to assess the impact of these shared rigor and reproducibility reporting recommendations comprehensively across the open access scientific literature. It is now possible to create a Rigor and Transparency Index (RTI) across journals that can be compared with current metrics such as the Impact Factor.

**Table 1:** Rigor Criteria with Applicable Guideline Source and Brief Description Listed

Entity Type	Source	What is This?
**Rigor Criteria (5 Total Points)**		

Institutional Review Board Statement^a^	MDAR	A statement (usually a single sentence) addressing IRB approval for biomedical research involving human subjects (or why IRB approval was not required)
*Example*: All human work was conducted under human subjects protocols approved by the Stanford Institutional Review Board (IRB), the University of Michigan UM-IRBMED, and the ethical committee of d’Ile de France II. *Example:* The trial was approved by the NRES committee London—South East.		
Consent Statement^a^	MDAR	A statement (usually a single sentence) addressing subject/patient consent in human research (or why consent was not required)
*Example*: Written informed consent was obtained from parents of all participating children and oral assent was obtained from 7-year-olds. *Example:* All infants were enrolled with informed parental permission under a protocol that was reviewed and approved by the Institutional Review Boards of the respective study sites.		
Institutional animal care and use committee statement	MDAR, ARRIVE	A statement (usually a single sentence) addressing IACUC ethical approval for research involving vertebrate organisms
*Example*: All animal experiments were performed in accordance with relevant guidelines and regulations and were approved by the University of Pennsylvania Institutional Animal Care and Use Committee (IACUC). *Example*: All animals used in this study were treated in accordance with UK animal (scientific procedures) legislation and under the appropriate project licenses, national and local ethical approval.		
Randomization of subjects into groups	MDAR, NIH, CONSORT, ARRIVE	Considered addressed when a statement describing whether randomization was used (e.g., assigning subjects to experimental groups, positions in a multiwell device, processing order, etc.)
*Example*: Animals were assigned to experimental groups using simple randomization. *Example*: Communication with schools, and elicitation of willingness to participate, was conducted before the village-level randomization took place.		
Blinding of investigator or analysis	MDAR, NIH, CONSORT, ARRIVE	A statement discussing the degree to which experimenters were unaware (or blinded) of group assignment and/or outcome assessment
*Example*: Responses were then scored by an experimenter blinded to injection condition and experimental cohort. *Example*: All the analysis was performed by a person unaware of the experimental question.		
Power analysis for group size	MDAR, NIH, CONSORT, ARRIVE	A statement addressing how (and if) an appropriate sample size was computed
*Example*: Sample size was based on estimations by power analysis with a level of significance of 0.05 and a power of 0.9. *Example:*Sample size calculation was done for the primary aim of this study, i.e., FMD, as reported previously.		
Sex as a biological variable	MDAR, NIH, CONSORT, ARRIVE	Reporting the sex of any and all organisms, cell lines, and human subjects
*Example*: Six healthy adult rhesus macaques (*Macaca mulatta*) of Chinese origin (4–8 kg, three males and three females, 4–8 years old) were inoculated intramuscularly (i.m.) with 1,000 pfu of EBOV Makona strain. *Example:* In each session, the behavior of each mother was recorded every 2 min.		
Cell Line Authentication^b^	MDAR, NIH	A statement detailing how the cell lines used were authenticated (e.g., short tandem repeat analysis). This is only required when cell lines are detected
*Example*: MOLM-14 cells were authenticated by STR profiling and flow cytometry. *Example:* All cell lines were obtained from ATCC, tested negative for mycoplasma, and their identity was verified by short tandem repeat analysis (Promega GenePrint 10 system).		
Cell Line Contamination Check^b^	MDAR, NIH	A statement addressing the mycoplasma contamination status of the cell lines used. This is only required when cell lines are detected
*Example*: All cell lines were obtained from ATCC and tested negative for mycoplasma contamination. *Example*: All cell lines were confirmed to be mycoplasma free using a PCR-based detection strategy with positive and negative controls.		

**Key Biological Resources (5 Total Points)**		

Antibody	MDAR, NIH, STAR, RRID	SciScore attempts to find all antibody entities within the methods section. “Identifiable” antibodies are reported with any metadata required to uniquely identify the antibody used such as vendor, catalog number, clone ID, batch number, or RRID
*Example*: ATF3 antibody (Santa Cruz Biotechnology) was used at 1:2000. Example: Slices were then washed (3x) and placed in PBS containing the following: 1% (vol/vol) normal goat serum, 1% (vol/vol) BSA, 0.25% (vol/vol) Triton X-100, and mouse monoclonal anti-5.8S rRNA, clone Y10b at 1:500 (Abcam, ab37144, RRID: AB_777714) overnight at 4°C.		
Organism	MDAR, NIH, RRID, STAR, ARRIVE	SciScore attempts to find all organism entities within the methods section. “Identifiable” organisms are reported with any metadata required to uniquely identify the organism used such as vendor, catalog number, or RRID
*Example (mouse)*: Adult (10–12 weeks; 25–30g) male C57BL/6 and TH-Cre mice were group-housed until surgery. *Example (fly)*: To generate PIP821bpΔ the following sgRNA was generated 5′-GCAGGAGGAGGTACAGCGGG-3′ and cloned into pU6-2-BbsI-gRNA (DGRC #1363) and then subsequently injected into w1118; vas-Cas9 (RRID:BDSC_51324, rainbow transgenics). *Example (fish):* The transgenic lines used in this study were Tg(kdrl:EGFP)s843 (Jin et al., 2005), Tg(lyve1b:dsRed2)nz101, Tg(lyve1b:EGFP)nz150 (Okuda et al., 2012), Tg(mpeg1:EGFP)gl22, Tg(mpeg1:Gal4FF) gl25 (Ellett et al., 2011), Tg(lyz:EGFP)nz117 (Hall et al., 2007), Tg(i-fabp:RFP)as200 (Her et al., 2004), Tg(UAS-E1b:nfsB-mCherry)c264 (Davison et al., 2007) and Tg(-8.mpx:KalTA4)gl28.		
Cell line	MDAR, NIH, STAR, RRID	SciScore attempts to find all cell line entities within the methods section. “Identifiable” cell lines are reported with any metadata required to uniquely identify the cell line used such as vendor, catalog number, or RRID
*Example*: The lung cancer cell line, H1299, was obtained from the American Tissue Culture Collection (Manassas, VA). *Example:*J774A.1 murine monocytes and macrophages (ATCC, number TIB-67) were cultured at 37°C in a humidified air/carbon dioxide (CO2) (19:1) atmosphere in RPMI medium supplemented with 10% (v/v) heat-inactivated fetal bovine serum, penicillin (100 IU/mL), streptomycin (100 μg/mL), and amphotericin B (250 ng/mL).		
Plasmid^c^	STAR, RRID	SciScore attempts to find all plasmid entities within the methods section. Plasmids were not used in this analysis
*Example*: The constructions were prepared using the vector *pSpCas9(BB)-2A-Puro (PX459) V2.0*, which was a gift from Feng Zhang (Addgene plasmid #62988; RRID: Addgene_62988). *Example:* For expression in HEK293 cells, INF2 was first subcloned into pGADT7.3 (BspEI/XmaI-XhoI) and then into pEGFP-C3 (EcoRI-SalI).		
Oligonucleotide^c^	STAR	SciScore attempts to find all oligonucleotide entities within the methods section. Oligonucleotides do not impact score and were not used in this analysis
*Example*: Activating Notch1 mutations in mouse models of T-ALL (*Blood* 2006,107:781–785), including one new oligonucleotide primer pair: Ex34B-f: 5′-GCCAGTACAACCCACTACGG-3′; Ex34B-r: 5′-CCTGAAGCACTGGAA-AGGAC-3′. *Example*: Primers used were GRHL2-1-424-F (TATATAGGATCCATGTCACAAGAGTCGGACAA), GRHL2-1-424-R (ATATAAAGATCTT-TTTCTTTCTGCTCCTTTGT), GRHL2-438-625-F (TAAATTAGATCTAAAGGCCAGGCCTCCCAA-AC), and GRHL2-438-625-R (TTATATGTCGACCTAGATTTCCATGAGCGTGA).		
Software Project/Tool	STAR, RRID	SciScore attempts to find all software tools within the methods section. “Identifiable” tools are reported with an RRID or are able to be uniquely identified through a distinct name/URL
*Example*: ImageJ was used to process and analyze raw images. *Example:* All simulations were performed using the NEURON simulation environment (Carnevale and Hines, 2006).		

SciScore was trained to recognize each of these criteria, with the training dataset size and classifier performance listed in Table S1. Example sentences recognized by SciScore are listed for each criterion, and we have underlined the entity that was likely to be recognized by the tool. These types of entities were annotated by curators to train individual classifiers.

a Institutional Review Board and consent statements are scored together as a block where detection of one or more of these entities will give the full point value for this section.

b Cell line authentication and contamination statements are only scored when a cell line is detected in the key resources table and they are scored together, either of these will provide the full points for this section.

c Entity type not used for analysis in the current paper.

## Results

### Classifier Performance Analysis

We obtained data from the PubMed Central (PMC, RRID:SCR_004166) Open Access (OA) subset, 1,578,964 articles (4,686 unique journals) and we analyzed the methods sections of these articles using SciScore. Of the 1,578,964 articles analyzed, 197,892 articles were considered not applicable (SciScore = 0). In total, 1,381,072 papers were scored, giving a score rate of 87.5% for articles with accessible methods sections. For information on the construction of individual SciScore classifiers, the reproducibility features detected by the tool (Table 1), example sentences that are found by the classifier (Table 1), raw classifier performance (Table S1), and classifier validation methods, please see the Transparent Methods section of this manuscript.

To validate the functioning of the complete tool, as opposed to individual classifiers (individual classifier performance is detailed in Table S1), we sampled two different groupings of 250 papers to determine the quality of our annotations and test our classifier performances (SciScore >0 and SciScore = 0; see Transparent Methods). For the 250 scored papers, the curator-SciScore agreement rate for each entity type is shown in Table S2. In every case, the entity type had an agreement rate above 80%; most were over 90%. The false-negative values and false-positive values for each entity type are listed in Table S1. The overall curator-SciScore agreement should represent additive probability if more than one sentence in the manuscript describes the item. For example, two cell lines are used in the average paper (Babic et al., 2019), so the probability of finding either is generally more likely compared with finding just one. In all cases, the agreement rate was measured above the raw classifier F1 rate as would be expected, except for software tools, which had an agreement rate that was lower than expected based on our previous training data (Table S1).

### Reproducibility Criteria over Time

#### Rigor Adherence and Key Resource Identification

We scored PMC data and grouped the data by journal and year showing the average SciScore, RTI. We also report the proportion of papers addressing specific rigor criteria and the proportion of uniquely identifiable resources. In total, only 8 papers in 1,578,964 received the maximum score of 10. Data are included in the supplemental file for this paper (Data S1; also found here). Summary data are presented to preserve author anonymity.

**Figure 1:**
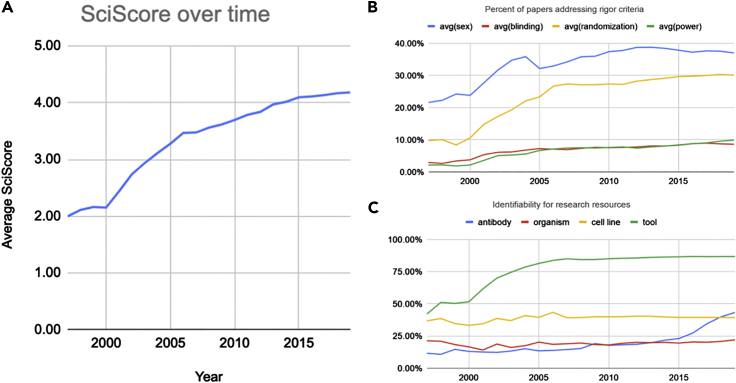
Overall Scores and Their Breakdown Shown between 1997 and 2019 (A) Average score of the dataset representative of the biomedical corpus showing a relatively steady increase over time. (B) Percentage of papers mentioning the use of sex, blinding, randomization of subjects, and power analysis. Sex and randomization have increased significantly, whereas blinding and power analysis have increased but are still at relatively low rates. (C) Percentages of key resources (antibodies, organisms, cell lines, and software tools) that are considered uniquely identifiable. Rates of software tools and antibodies have increased, whereas organisms and cell lines have remained relatively stagnant. Data underlying these graphs are available in Data S2.

Between 1997 and 2019, the average annual score has more than doubled from 2.0 ± 0.9 to 4.2 ± 1.7 (Figure 1A). This increase in score coincides with increased levels of both rigor criteria inclusion and key resource identifiability. The year 1997 was chosen as the starting point as years prior to this did not meet our minimum required sample size (e.g., 1996 [n = 73] versus 1997 [n = 1,024]). For rigor criteria inclusion, adherence levels largely increased for the following criteria: sex (21.6%–37.0%) and randomization of group selection (9.8%–30.1%). Levels of inclusion of statements about blinding (2.9%–8.6%) and power analysis (2.2%–9.9%) increased but remained relatively low (Figure 1B). For key resource identifiability, antibodies (11.6%–43.3%) and software tools (42.1%–86.7%) were increasingly found to be uniquely identifiable in the methods section, whereas organisms (21.1%–22.0%) and cell lines (36.8%–39.3%) remained at low levels of identifiability (Figure 1C).

The PubMed Central papers that we analyzed might not be preclinical studies, in which case many of the rigor criteria may not apply. For example, some chemistry papers were certainly analyzed, but we cannot be certain what percentage of the papers analyzed fit these criteria. Therefore, we reran the analysis with papers that used vertebrate animals, which should exclude most clinical or chemistry studies and the proportion of papers using the criteria should be more reflective of the animal, pre-clinical literature where rigor criteria are most relevant. Papers containing IACUC statements in 2018, the most recent full year, comprised about a quarter of the OA subset (51,312 of 208,963). These animal papers showed the following rates of rigor criteria: sex 55.82% (compared with 37.54% in the total set), blinding 12.33% (8.74%), randomization 36.26% (30.30%), and power 7.34% (9.57%). Of 62,652 organisms detected in the total literature, 51,134 were represented in the subset. Identifiability of organisms was 21.71% versus 20.81% in the total set. These numbers suggest a trend in that the vertebrate animal subset of the literature is somewhat more transparent than the total literature especially when looking at sentences describing the sex of the animal, group selection criteria and blinding, however, it remains far from ideal.

#### Antibody Identification

**Figure 2:**
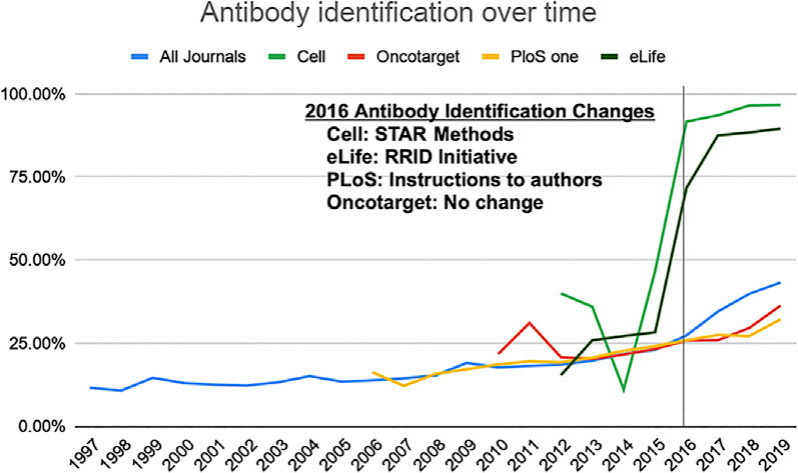
Percentage of Antibodies That Are Able to Be Uniquely Identified Shown by Journal with the Overall Trend across the Biomedical Literature Shown in Blue A significant improvement can be seen starting in 2016 for *Cell* and *eLife* when STAR methods formatting and RRIDs were first implemented in their respective journals contributing to a noticeable improvement in antibody identifiability for the entire biomedical literature. Data underlying this graph are available in Data S3.

In just the last few years, antibody identification has made considerable improvements going from the least identifiable key resource to the second most identifiable one, although antibody identification still remains under 50% overall. Some journals have made significant changes, leading to a more dramatic improvement compared with others (Figure 2). For example, *Cell*, a STAR methods journal, improved their antibody identifiability rates from 11.1% to 96.7% from 2014 to 2019. *eLife*, a participant in the RRID Initiative, increased their antibody identifiability rates from 27.2% to 89.6% from 2014 to 2019. On the other hand, *Oncotarget* (21.6%–36.4%) and *PloS One* (22.7%–32.2%) have each improved, but their absolute rates remain relatively low, with each falling below the overall average during that time frame (21.8% in 2014; 43.3% in 2019).

Table 2 shows the top 15 journals with the highest antibody identification rates for 2019 along with the number of antibodies detected in each. Seven (46.7%, *Cell Stem Cell*, *Immunity*, *Cell*, *Molecular Cell*, *Developmental Cell*, *Cell Metabolism*, *Current Biology*, and *Cell Reports*) have implemented the STAR methods reagent reporting format. Fourteen (93.3%, see Table 2) participated in the RRID Initiative and continue to enforce the use of RRIDs as of 2019. Therefore, these two drivers (STAR methods implementation and RRID Initiative participation) appear to have meaningfully contributed to improving the rate of identifiability in a majority of the best antibody identifying journals.

**Table 2:** Top 15 Journals Sorted by Percent of Antibodies that Were Identifiable in 2019

Papers Analyzed	Year	Journal	Antibodies Detected	Identifiable	% Identifiable
13	2019	*Cell Stem Cell*^a^	242	236	98%
16	2019	*Immunity*^a^	430	419	97%
24	2019	*Cell*^a^	362	350	97%
42	2019	*Molecular Cell*^a^	548	524	96%
11	2019	*ASN Neuro*^a^	84	80	95%
13	2019	*Developmental Cell*^a^	123	117	95%
12	2019	*Cell Metabolism*^a^	181	171	94%
17	2019	*Current Biology*: CB^a^	132	123	93%
14	2019	*The Journal of Neuroscience*^a^	91	84	92%
11	2019	*Particle and Fibre Toxicology*	11	10	91%
240	2019	*Cell Reports*^a^	1,458	1,312	90%
491	2019	*eLife*^a^	2,476	2,218	90%
75	2019	*eNeuro*^a^	344	308	90%
18	2019	*BMC Biology*^a^	62	55	89%
21	2019	*Journal of the Endocrine Society*^a^	69	58	84%

For this analysis, there were 682 journals in which more than 10 antibody containing articles were accessible in our dataset.

a Indicates participation by the journal in the RRID initiative as of 2019. The complete dataset is available as Data S7.

#### Cell Line Identification and Authentication

All cell lines should be authenticated according to the international cell line authentication committee (ICLAC) guidelines because cell lines often become contaminated during experiments (Capes-Davis et al., 2010). Authentication of cell lines is usually accomplished by short tandem repeat (STR) profiling. This procedure is recommended at the outset of the experiment, at the conclusion of the experiment, and at a random time during the experiment. If this important control is completed, it should be stated in the manuscript. Similarly, authors should also test whether mycoplasma has contaminated their cell lines. For our purposes, we treated checking for mycoplasma contamination and authentication assessment like STR profiling as evidence that authors checked at least some aspect of cell line authenticity. Table 3 shows the journals that have the highest rates of authentication or contamination and the identifiability of cell lines in those journals. The percent of authentication is calculated as the percent of papers that contain a contamination or authentication statement in papers where at least one cell line is found.

**Table 3:** Top 15 Journals Sorted by Percent of Cell Line Authentication (Authentication or Contamination) that Were Identifiable in 2019.

Papers Analyzed	Year	Title	Cell Lines Found	% Identifiable	% Authentication
278	2019	*Elife*	849	55%	71%
11	2019	*Nature Microbiology*	39	33%	64%
83	2019	*Oncogene*	302	41%	54%
23	2019	*Journal of Cell Science*	87	31%	52%
27	2019	*Nature*	85	47%	52%
33	2019	*Oncogenesis*	95	36%	52%
14	2019	*Breast Cancer Research*	86	35%	43%
29	2019	*EMBO Molecular Medicine*	65	38%	41%
17	2019	*Disease Models & Mechanisms*	23	43%	41%
17	2019	*EMBO Reports*	42	36%	41%
18	2019	*Cell*	67	36%	39%
178	2019	*BMC Cancer*	485	40%	38%
37	2019	*Molecular Cell*	157	37%	38%
14	2019	*Therapeutic Advances in Medical Oncology*	48	44%	36%
946	2019	*Nature Communications*	2,601	38%	34%

There were 2,280 journals in which more than 180,316 articles and more than 388,337 cell lines were accessible in our dataset. The complete dataset is available in Data S8.

#### *Nature*'s Rigor Adherence

**Figure 3:**
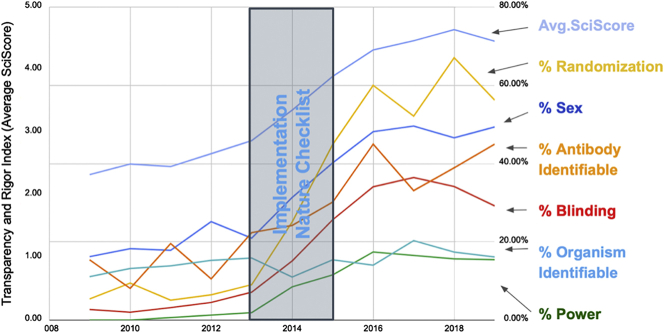
Analysis of Rigor Criteria for the Journal *Nature* The right axis represents the percentage of papers that fulfill a particular criterion. The left axis represents the average SciScore. The figure shows that, during and after the implementation of the *Nature* checklist, the average SciScore as well as all measures except for organism identifiability have improved markedly. Although scores were increasing before the checklist implementation, the checklist appears to quickly boost numbers. Data underlying this graph are available in the (Data S1 and S4, https://scicrunch.org/scicrunch/data/source/SCR_016251-1/search?q=∗&l=∗).

Checklists may assist authors in finding aspects of their manuscript that were not addressed, but until now it has been very difficult to determine if these checklists are effective. Most studies that addressed this issue looked at a relatively limited sample of journal articles (Leung et al., 2018). We consider below a use case, in which the implementation of a checklist system appeared to be effective in improving the number of rigor criteria addressed by authors. In 2013 to 2014, *Nature* made a significant push with authors to address rigor criteria. We plotted the average score along with its components over this period (Figure 3) and found that the average score rose by nearly two points over just a few years. This is based largely on a concomitant rise of authors addressing blinding, randomization, and sex of subjects. To a smaller degree, antibodies became more identifiable and power analysis was described in a larger proportion of papers. In stark contrast, the *Proceedings of the National Academy of Sciences of the United States of America* (*PNAS*), which put out several reports advocating for the need for increased rigor (Allison et al., 2018), showed no sizable change in composite score: 3.33 in 2015 to 3.42 in 2019.

#### A Comparison of the Rigor and Transparency Index with the Journal Impact Factor

**Figure 4:**
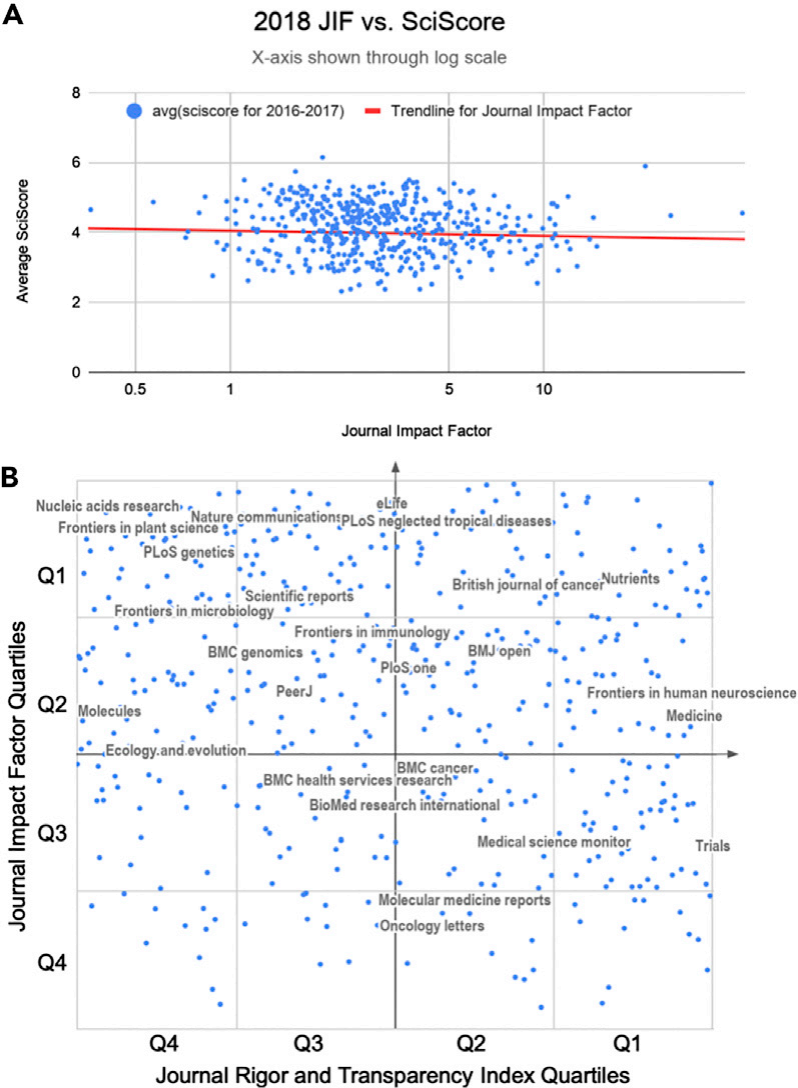
Average Journal SciScore between 2016 and 2017 as a Function of the Journal Impact Factor for 2018 (Data from Published Papers from 2016 to 2017) Data from 490 journals are shown in each graph. (A) A comparison between the raw JIFs and Rigor and Transparency Index is shown. The correlation coefficient is calculated using the formula for Spearman's rank-order correlation (R_s_ = −0.1102253134). (B) A comparison between JIF percentiles and SciScore percentiles is shown. The axes are labeled with quartiles; top quartile is Q1. For presentation purposes only, using Google Sheets with journal names as centered data labels, we chose the top 45 journals by the number of articles included and then we removed labels that were overlapping until we were left with 25 labeled journals, shown above. All 490 journals, for which we had sufficient data in the open access literature to compare to the Journal Impact Factor, are presented in (Data S5). Correlation values were calculated using the formula for Spearman's rank-order correlation, the line is not shown (R_s_ = −0.1541069707).

In total, we included data from 490 journals (totaling 243,543 articles) for the Journal Impact Factor (JIF) versus Rigor and Transparency Index (average SciScore) comparisons. The comparison between the raw JIFs and the Rigor and Transparency Index (Figure 4A) showed a slight negative relationship; however, the correlation coefficient (R_s_ = −0.1102) suggests that this is not a significant relationship. Similarly, the JIF percentile versus SciScore percentile relationship showed no significant correlation (R_s_ = −0.1541; Figure 4B).

## Discussion

In this study, we introduce an automated tool, SciScore, that evaluates the materials and methods sections of scientific papers for adherence to several key reporting guidelines introduced by funding agencies and journals over the past decade. The reason that we focus on the methods section is that the methods section is the bedrock of the scientific study on which all results and conclusions rest and is also almost completely knowable. In this section of the scientific paper there are absolute facts, such as which reagents were used, how long a rat was exposed to a drug, or how many zebrafish were male or female. This also enables us to not consider statements that authors make about reagents that they did not use because the methods section of a paper typically states where a reagent was purchased and how it was treated. Because the tool is automated, it provides us the opportunity to look at overall trends in adherence to these guidelines across the breadth of the scientific literature for the first time at an unprecedented scale.

### Analysis of Reporting Trends

Since the early 2000s, there have been multiple calls to improve scientific reporting and increase the specificity within methods sections because of irreproducible research (Ioannidis, 2005; Open Science Collaboration, 2015). In 2007, Sena and colleagues used meta-analysis to assess the presence of various rigor criteria in the scientific literature about different diseases (Sena et al., 2007). Although we were not able to exactly replicate those findings, our results can be compared. In their study, a human curator scored the presence of rigor criteria in 624 papers, a tremendous amount of human effort. Rigor criteria prevalence were broken down into disease groups, including stroke, multiple sclerosis, and Parkinson's disease. In this set of the literature, authors addressed randomization of subjects into groups between 1% and 10% of the time depending on the disease group. In our data, randomization is addressed in 8%–27% of papers between 1997 and 2007 with a steady rise over time. In the Sena paper, blinding was addressed 2%–13% of the time, whereas our data show a range of 3%–7% of papers. Power analysis calculations were not detected in any study by Sena and our data show a detection rate of 2%–7%. However, our data include pre-clinical and clinical studies, whereas Sena's study only included the former, making a direct comparison a little more tenuous. Although reasonable people may argue that different techniques were used in performing these studies, including study selection and the criteria for inclusion, there is a striking similarity in this very cursory comparison suggesting that the overwhelming majority of studies published in 1997–2007 did not address randomization, blinding, and power analysis. This result is not entirely surprising given that these factors were specifically identified as leading to problems with reproducibility and were therefore targeted in the reporting guidelines that emerged after this period.

Since 2007, there has been a steady improvement in rigor inclusion and key resource identifiability rates across the literature. Between 1997 and 2019, the average score of biomedical research has more than doubled indicating an improvement in the transparent reporting of scientific research. However, it is difficult to assign causality. Although the checklist implemented at *Nature* has clearly been well executed (see Figure 3), in general, guidelines and checklists have been shown to be relatively ineffective at improving the reporting tendencies of authors; because of this, we highly doubt these improvements are entirely due to the presence of checklists and guidelines (Kilkenny et al., 2009). However, that is not to say that these guides are useless. We believe these guides help disseminate knowledge to authors, providing them with good focal points for where they should put forth effort in order to improve the reporting of their research, and although efforts such as the ARRIVE guidelines initially remained relatively unsuccessful in changing author behavior, there was eventual improvement (Figure 1). Given our current dataset, we can state that these reporting improvements appear to be occurring across biomedicine in general, suggesting that they may be due in part to an increase in awareness of the importance of reporting on good scientific practice.

Although there are many causes contributing to the complex issue of scientific irreproducibility within biological research, none have been more vilified than the antibody (Baker, 2015). As one of the most prevalent tools in modern-day biological research, they represent an easy target raising the ire of disgruntled scientists as they are known in many cases to display a high level of variability between sources (Voskuil, 2017). These issues, however, cannot be discovered in most papers because even today, these reagents are not usually cited in a way that makes it easy to even understand which antibody has been used. Antibodies have long been one of the least identified resources (Figure 1C; Vasilevsky et al., 2013). In 2013, Vasilevsky et al. analyzed 238 manuscripts to measure the identifiability of a variety of resources including antibodies. Comparing the Vasilevsky antibody results to our current analysis, we found that antibodies are identifiable less often. For 2013, Vasilevsky found that ~45% of antibodies are identifiable, whereas our algorithm found it to be ~20% (in 2013). This discrepancy can likely be attributed to differences in criteria used, the exact papers analyzed, and the size of the sample. For each antibody, Vasilevsky looked in all vendor catalogs and searched for the name of that antibody, if the vendor search resulted in only one antibody, it was considered identifiable. This presents a bit of a best-case scenario for antibody identification as similarly named antibodies may be added to a company's inventory in the future or an antibody may simply have its name altered over time. Our algorithm relies on the presence of either a catalog number or an RRID for identifiability, which are far more stringent. Although catalog numbers may still be quite imperfect for identifiability (Voskuil, 2014), they are nevertheless far more stable than product names in a vendor's catalog. Additionally, RRIDs are significantly more stable than either a product's name or its catalog number as they are meant to serve as a sort of unique product code (UPC) that transcends any of these superficial changes.

Data about software tools may be subject to more significant recency effects than other resources. This is partially because SciScore can detect that a word or phrase is the name of a software tool, but to be considered identifiable the tool must have accessible metadata. It is relatively more likely that tools in common use are more identifiable than tools that may have been used over a short period of time. Despite this, we still feel that SciScore has captured a majority of the mentioned software tools. This is because software tool mentions appear to follow the 80/20 rule where roughly 80% of the mentions are related to 20% of the tools (Ozyurt et al., 2016).

Our analysis of the antibody data clearly demonstrates that some journals, which enforce RRIDs, have dramatically higher rates of identifiability (>90%) than the average journal (~40%) (see Table 2). Enforcing the use of RRIDs is not an effortless exercise; we understand from personal communications with the editors of the *Journal of Neuroscience* that authors are asked to identify their research resources three times during the publication process (Marina Picciotto, personal communication). As a group, Cell Press journals in particular have done an exemplary job in identifying antibodies. By requiring a STAR table (a standardized key resources table), Cell Press has created a way to make key resource identification highly visible (Marcus, 2016). This is especially important because it appears that they are not only moving their authors to change behavior but that these changes in behavior are also spreading as evidenced by a fairly dramatic overall shift in identifiability since 2016. We do not know why this spread is occurring, but in seemingly unrelated journals that did not change policies with regard to antibody identification there are more well-identified antibodies. Some of this could be explained as journals that enforce policy have high rejection rates and those authors end up in another journal with well-identified antibodies. It may also be that authors have been frustrated for so long trying to track down antibodies that when they hear about a way to change the current practice, they embrace the change.

### Comparison of the RTI with Other Metrics

Through the use of a vastly different performance indicator than what is currently used (SciScore as opposed to JIF), we have created a method to score journals that is very different from the impact factor. The Rigor and Transparency Index lists journals with their composite scores and rates of inclusion for rigor adherence and resource identifiability. Our analysis indicates that there is no correlation between a journal's impact factor and the Rigor and Transparency Index, which highlights the uniqueness of the RTI as a metric independent of research popularity.

Researchers have pointed out various problems with measuring journals based on the JIF (Davis, 2019; Parish et al., 2019; Rawat, 2014; Vanclay, 2011). Many of these arguments are valid in that they point to this single number as an “outdated artifact” that improperly impacts how we view research. The most important underlying problem with the JIF, in our opinion, is that it measures popularity (number of citations) and not the quality of the work. Although some may argue that we are simply switching one problematic metric with another, this is a gross oversimplification.

The Rigor and Transparency Index differs from the JIF in that it is based on known problem areas linked to the inability to reproduce a study. Although the composite number for any given study is likely nearly meaningless (an 8 is not demonstrably better than a 7, for example), it is very difficult to argue that reagents used in a study should not be referenced in such a way as to easily identify them. It is also true that all means of reducing investigator bias, such as blinding, are not possible in all experimental designs, especially during the conduct of certain experiments. However, it is difficult to argue that addressing investigator bias is a waste of time; indeed, investigators surveyed by *Nature* overwhelmingly state that the MDAR checklist, which covers bias, was helpful to their reporting of research (Hawkins, 2019). Investigator bias can creep into any scientific discipline and has been shown to artificially inflate effect size in stroke research (Sena et al., 2010), but these effects have been well understood since the 1960's (Rosenthal and Fode, 1963) and have informed the practice of clinical trials. Although currently it is nearly impossible to determine if authors are addressing rigor criteria appropriately, an automated metric like the RTI can still be used to increase transparency in reporting, allowing readers to make better, more informed decisions regarding an experiment's reproducibility. The fact that most authors largely ignore these criteria shows that investigator bias is not “on the radar” of many researchers as they report findings. By elevating the visibility and importance of rigor and transparency through the use of something like the RTI, we believe we can improve the quality of scientific reporting and, thus, improve scientific reproducibility. In more general terms, we believe that research that completely and transparently reports its reagents and methods is likely to be better than research that does not. We therefore argue that a study that scores 8 or 9, which will necessarily address investigator bias and uniquely identify most resources, is better than a study that does not address these and scores 2 or 3.

We also examined the potential relationship between the RTI and the newly released 2019 TOP Factor using data from 31 journals where the ISSN numbers match between TOP and RTI. Similarly to the JIF, there was no correlation between the two metrics (Pearson's correlation). Ideally, there would be some correlation between two metrics of rigor, and perhaps once more data are released for TOP, this could be the case in the future. However, although both the TOP Factor and the RTI generally try to quantify rigor and transparency in journals, they go about it in two different ways. The TOP Factor is calculated based on a journal's reporting best practice guidelines, whereas RTI is calculated based on whether or not authors within a journal actually follow some of these guidelines at the individual article level. We know from previous works that guidelines are rarely followed (Bandrowski et al., 2015; Hair et al., 2019; Kilkenny et al., 2009; Leung et al., 2018), so although a journal may have very strict guidelines, the current lack of relationship suggests that authors do not change their behavior accordingly.

Future goals of the RTI include improving the detection of power calculations and the authentication and contamination of cell lines. These classifiers worked relatively poorly compared with the other parts of SciScore. In addition to expanding datasets, we plan to add additional criteria (e.g., inclusion/exclusion criteria and replication information) in future versions of SciScore to improve our coverage of entities required to complete the MDAR checklist.

The creation of the Rigor and Transparency Index provides both a short hand for how a journal is doing and a much more detailed picture of the current state of rigor and transparency practices. It can point each journal to significant problem areas that are addressable in future publications. It also provides journals and funders the ability to monitor the impact of their policies regarding rigor and reproducibility. The RTI can bring attention to the importance of sound scientific practices.

### Limitations of the Study

A limitation of the study is that the OA subset represents only a fraction of the total biomedical literature and as a result must be considered a biased subsample. First, because these data consist of only full text accessible, open-access papers or copies of closed access papers supported by the NIH with licenses permissive for text mining, some journals may not be represented. Second, most of the data available through PMC are from the past 5–10 years, whereas PubMed contains a significant number of articles that date back 30 to 40 + years. Because of these concerns, we concede that PubMed, with 30.37 million articles as of November 27, 2019, is only partially represented in the portion of PubMed Central accessible for text mining. As a result of this differential, we cannot be certain that the text-mining accessible papers in PubMed Central are completely representative of the totality of biomedical literature. However, given that PubMed is our best guess at the totality of the biomedical literature, then it stands to reason that a sample of this magnitude should be a reasonable representation of the total, especially in the more recent years. Journals that are not represented in this dataset are either those that are unavailable as open access or unavailable under a text-mining allowed license.

Another limitation is that any information that is not included in a text-mining accessible methods section is omitted from review by SciScore. One of the most glaring omissions from our dataset is the journal *Science*. There are several reasons why this may be the case, the most likely being that their articles are not included in the OA subset because of restrictive licenses. Another potential reason is that their articles' methods sections are simply unreadable to SciScore. Since *Science*'s format is highly abbreviated, the methods section tends to be pushed into the supplementary materials where it is likely to be formatted as an image (PDF) rather than as text. To a text mining algorithm like SciScore, a supplementary PDF is effectively invisible. If we were to attempt to score these papers manually instead, it would take roughly 1,500 h or 187 days of nonstop curation to score the 18,000 *Science* papers in PMC assuming each paper took 5 min. As a point of reference, SciScore averaged about 2 s per paper, so *Science* would take 10 h on a single machine (the 1.6 M articles were processed in about 6 weeks). The biomedical literature is produced at a rate of about 2 million articles per year, a rate that long ago exceeded the ability of any human to read, much less deeply understand the content. We expect that scientists will need a helping hand from some form of robot that can pre-digest content, but to be effective, this robot will need access. It would be a real shame if the flagship journals were not represented in this new paradigm (Carpenter, 2017).

As previously mentioned, SciScore was originally developed to score biomedical research articles and the training data consist entirely of sentences from the methods sections of papers. This might be viewed as problematic given that PMC contains non-biomedical research journals where SciScore's scoring criteria may not be deemed fully relevant. We attempted to mitigate this by excluding papers that had scored a 0. Papers without any criteria found (SciScore = 0) were not included in our analysis as we considered SciScore's criteria “not applicable” in these cases. In future versions of SciScore, we plan on implementing additional criteria and conditional scoring (e.g., only expecting an IACUC when vertebrate organisms are detected) to be able to more accurately score a wider range of papers.

The use of SciScore versus individual classifiers differs, where SciScore in total performs a little better than expected if one were to consider only the individual classifier performance, as evidenced by the agreement between the tool and curator (Table S1). Some of this can be explained by the fact that research resources are often used in multiples, and these may be described several times, increasing the ability of SciScore to pick up at least one of the mentions. Another difference is that SciScore triggers a second pass with less stringent criteria when some features, such as RRIDs, are detected by regular expression in the text. This allows for a higher recall than expected by the raw classifiers in cases where we are certain that a key resource is being described, without the downside of reduced precision.

### Resource Availability

#### Lead Contact

Further information and requests for materials should be directed to and will be fulfilled by the lead contact, Dr. Anita Bandrowski (abandrowski@health.ucsd.edu).

#### Materials Availability

This study did not generate new unique reagents.

#### Data and Code Availability

Code for retrieving and pre-processing XML data from the OA subset was previously published and is open source (https://github.com/SciCrunch/resource_disambiguator). Owing to the proprietary nature of SciScore, we cannot release its full source code. However, the resource disambiguator (RDW) mentioned above uses the same basic technology, a conditional random field-based named entity recognizer, which is directly used as part of SciScore. All RDW code is available. SQL statements (version hash from Open Science Chain RRID:SCR_018773; https://portal.opensciencechain.sdsc.edu/data/osc-5837f83f-31ab-426f-b8cc-84c7b9ec542a) and the Google spreadsheets (version hash https://portal.opensciencechain.sdsc.edu/data/osc-3c6555f9-9e55-4c9b-932a-82b799d6b0d4) used for analysis can be found in the supplemental materials provided. Summary data for each journal are provided through the supplemental files and have been made available via SciScore website (https://sciscore.com/RTI; RRID:SCR_016251). Data from individual papers from the OA subset will be made available upon request to researchers, but is considered sensitive because low scores assigned to published papers may be seen as negatively impacting scientists, without giving them the ability to respond to criticism or providing the same “criticism” for closed access publications. However, a limited number of individual papers can be submitted free at sciscore.com, and we encourage researchers to test their manuscripts for themselves.

## Methods

All methods can be found in the accompanying Transparent Methods supplemental file.

## Supplemental Information

Document S1. Transparent Methods, Figures S1, and Tables S1–S3

Data S1. All RTI Data Sorted by Journal by Year, Related to Figures 1–4

Data S2. RTI Data on the OA Subset Showing the Overall Scores and Their Component Breakdowns Shown between 1997 and 2019, Related to Figure 1

Data S3. RTI Data on the OA Subset Showing the Rates of Identifiability for Antibodies across the Entire Scored Literature, Cell, Oncotarget, PLoS One, and Elife, Related to Figure 2

Data S4. RTI Data from the OA Subset Showing the Rates at Which Rigor Criteria was Addressed for the Journal, Nature, Related to Figure 3

Data S5. SciScore Average Journal Data for 2016–2017 and Journal Impact Factor Data for 2018 (Representing data from Papers Published between 2016 and 2017), Related to Figure 4 Data are from 490 journals.

Data S6. RTI Data Showing the 50 Journals with the Highest Number of “Not Applicable” Scored Papers, Related to Data S1

Data S7. RTI Antibody-Related Data Broken Down by Journal and Year Ordered by the Rate of Antibody Identifiability, Related to Figure 2 and Table 2

Data S8. RTI Cell Line-Related Data Broken Down by Journal and Year Ordered by the Rate of Cell Line Authentication, Related to Table 3
