# Supplementary material for: The Rigor and Transparency Index Quality Metric for Assessing Biological and Medical Science Methods
Source: iScience. 2020 Oct 20;23(11):101698. doi: 10.1016/j.isci.2020.101698 (PMC7644557; doi:10.1016/j.isci.2020.101698)
Supplement: Document S1. Transparent Methods, Figures S1, and Tables S1–S3 [file mmc1.pdf]

iScience, Volume *23*

Supplemental Information

The Rigor and Transparency Index Quality Metric for Assessing Biological and Medical Science Methods

Joe Menke, Martijn Roelandse, Burak Ozyurt, Maryann Martone, and Anita Bandrowski

Supplemental Data Items

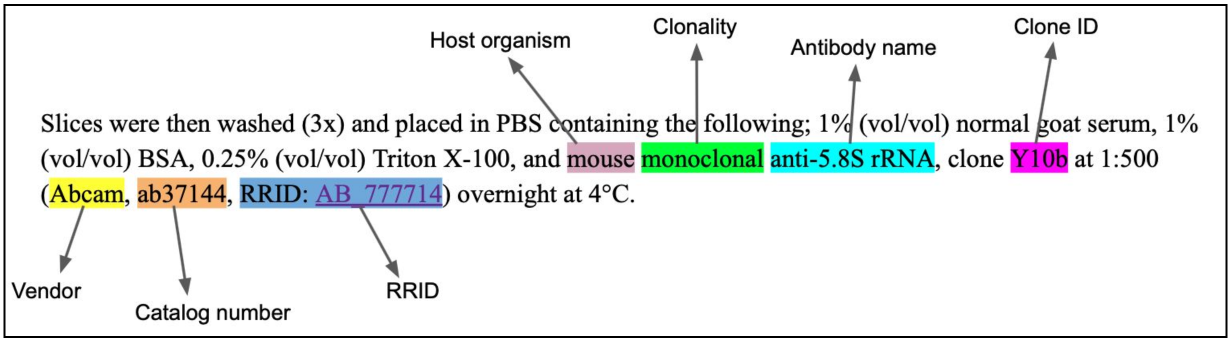

Figure S1: A visualization of the different components of an antibody that are part of the training set for SciScore. Related to Table 1.

Table S1. Individual Classifier Performance for Named-Entities. Training set size is shown as the # of entities, which represents the total number of entities tagged by our curators as either positive or negative and # of sentences, which represents the total number of sentences containing positive and negative examples as well as some sentences without any entities used in both training and testing. Related to Table 1.

**Entity Type**	**F1**	**Precision**	**Recall**	**Training Set Size** (# of entities/# of sentences)
	Mean ± SD	Mean ± SD	Mean ± SD	
**Rigor Criteria (5 total points)**				
Institutional Review Board Statement	81.41 ± 3.62	84.45 ± 5.26	79.57 ± 8.83	340/78,170
Consent Statement	94.75 ± 1.68	96.29 ± 2.42	93.38 ± 3.63	373/78,170
Institutional Animal Care and Use Committee Statement	81.30 ± 4.20	89.30 ± 4.60	74.89 ± 6.12	591/78,170
Randomization of subjects into groups	83.05 ± 3.04	80.25 ± 5.05	86.45 ± 4.64	368/52,945
Blinding of investigator or analysis	78.96 ± 12.38	77.74 ± 17.16	81.79 ± 10.32	183/52,945
Power analysis for group size	64.45 ± 29.37	73.74 ± 34.13	59.50 ± 26.91	81/52,945
Sex as a biological variable	88.32 ± 3.91	87.94 ± 6.03	88.93 ± 3.52	862/52,945
Cell Line Authentication	54.08 ± 11.88	85.70 ± 10.78	41.15 ± 12.82	155/14,792
Cell Line Contamination Check	91.70 ± 5.24	93.35 ± 7.15	90.65 ± 7.05	151/14,792
**Key Biological Resources (5 total points)**				
Antibody	78.94 ± 2.62	86.89 ± 3.78	72.46 ± 3.20	16,772/53,216
Organism	66.05 ± 4.70	79.91 ± 6.28	56.64 ± 5.75	4,439/45,500
Cell Line	70.07 ± 5.95	86.48 ± 3.27	59.34 ± 8.03	1,763/45,500
Plasmid [a]	79.62 ± 3.35	92.53 ± 3.80	70.09 ± 4.85	2,568/63,400
Oligonucleotide [a]	83.03 ± 9.05	95.28 ± 3.13	74.94 ± 13.90	1,893/63,400
Software Project/Tool	89.03 ± 0.90	92.49 ± 2.08	85.84 ± 1.10	10,161/19,002

a. Entity type not used for analysis in the current paper.

Table S2: Rates of false negatives, false positives, and overall agreement based on manual analysis of 250 scored papers (SciScore > 0) from our dataset. The curator generated data was considered always correct. Thus a false positive is when SciScore finds an item where the human curator did not. Agreement constitutes a much broader definition than Table S1. Here, agreement means that both the curator and SciScore found an item in the manuscript. If, for example, there are two sentences describing sex of subjects and the tool found one, while the curator found another, it would still be considered agreement. When considering key resources like antibodies or cell lines, authors tend to describe these in several sentences. Therefore even when the recall from Table S1 is 70%, recall of finding either of 2 sentences is over 85%. Related to Table 1.

Entity Type	False Positives		False Negatives		Overall Agreement	
	Size (#)	Rate (%)	Size (#)	Rate (%)	Size (# agreed)	Rate (%)
**Rigor Criteria**						
Institutional Review Board Statement or Consent Statement	11	4.4	3	1.2	236	94.4
Institutional Animal Care and Use Committee Statement	7	2.8	15	6.0	228	91.2
Randomization of subjects into groups	16	6.4	8	3.2	226	90.4
Blinding of investigator or analysis	2	0.8	7	2.8	241	96.4
Power analysis for group size	12	4.8	6	2.4	232	92.8
Sex as a biological variable	5	2.0	20	8.0	225	90.0
Cell Line Authentication or Contamination Check	12	4.8	0	0.0	238	95.2
**Key Biological Resources**						
Antibody	2	0.8	3	1.2	245	98.0
Organism	3	1.2	7	2.8	240	96.0
Cell Line	6	2.4	4	1.6	240	96.0
Software Project/Tool	8	3.2	41	16.4	201	80.4

Table S3. STAR Key Resources Table

REAGENT or RESOURCE	SOURCE	IDENTIFIER
Deposited Data		
Rigor and Transparency Index	This paper	https://sciscore.com/rti/
Software and Algorithms		
PubMed Central	N/A	https://www.ncbi.nlm.nih.gov/pmc/ RRID:SCR_004166
SQLite	N/A	https://www.sqlite.org/index.html RRID:SCR_017672
Journal Citation Reports	N/A	https://jcr.clarivate.com/JCRLandingPageAction.action RRID:SCR_017656
Clarivate Analytics	N/A	https://clarivate.com RRID:SCR_017657
Google Sheets	N/A	https://www.google.com/sheets/about/ RRID:SCR_017679
Open Science Chain	N/A	https://opensciencechain.org RRID:SCR_018773
SciScore	This paper	https://sciscore.com RRID:SCR_016251

Transparent Methods

Text mining the open access subset of PubMed Central

For this study, we downloaded and processed all open access literature available through PubMed Central (PMC, RRID:SCR_004166) in September of 2019. In total, we obtained data from 1,578,964 articles from 4,686 unique journals. The PMC Open Archives Initiative (PMC-OAI) was used to initially download the PMC Open Access subset (OA subset) as directories (one per journal named by the journal's standard abbreviation) allowing for a clear differentiation of each journal. Articles only available as PDFs were not included in the OA subset, and were therefore excluded from our analysis. In addition, abstract-only articles and articles without a methods section were also excluded from our analysis because the reporting criteria are generally included only in the materials and methods. We limited our analysis to journals that had published more than 10 papers for any given year available in the OA subset.

In order to create the dataset used for our analysis, the OA articles were fed through the named-entity recognition classifiers. SciScore currently uses 6 core named-entity recognition classifiers recognizing 15 primary entity types (see Table 1). In Table 1, a complete list of the primary entity types detected are shown along with their source(s) and a brief description. Criteria were generally chosen based on a variety of factors including our previous work (Grethe et al., 2016), feasibility (Can we identify the criteria using NER? How many examples will be needed to achieve high F1?), and the criteria's suspected impact on reproducibility (How many major guidelines/checklists does the criterion appear? Are other researchers voicing similar concerns?). It should be noted that SciScore is a long-term endeavour, thus the criteria presented here should be viewed as an initial set.

The classifiers (sequence taggers) use conditional random field (CRF) based algorithms to detect a variety of entity types (Lafferty et al., 2001). Each of these was validated using precision and recall as well as their harmonic mean, F1. The values for each entity type are listed in Table S1. Each classifier component was trained and tested separately for precision and recall using human curated data. The curator labeled each entity type within tens of thousands of example sentences using the smallest word chunk that was still informative. However, not all classifier components are visible in the composite tool result (see paragraph about antibodies below).

If the curator and algorithm did not have complete agreement with regard to the entity in our training dataset, it was considered a miss e.g., [anti-5.8S] vs. [anti-5.8S rRNA]. For rigor criteria (e.g. consent statements or cell line authentication statements), named entity recognition is used to identify words or word phrases that consistently appear in sentences of interest. In these cases, we report sentences rather than the individual entities within, although, we calculated F1 rates the same for each entity type where the exact entity had to be detected by SciScore with 0 edit distance to be considered a match even if the entity was found in the correct sentence. As a result, the classifier performances listed in Table S1 can be considered conservative estimates.

We tested these values using 10-fold cross-validation where 90% of the human curated data was used as training and 10% was used as the test. The final value comes from a mean of all 10 training trials. If the F1 was determined to be below the desired 70% threshold for key resources, we attempted to increase the training dataset size. Training sets contain sentences from the complete methods sections of published papers. Annotations were made using a NER curation tool (created by B.O.) that inserts XML snippets into XML training files. We did not set a minimum F1 threshold for our rigor classifiers as training data was far more difficult to locate for certain criteria, e.g., power analysis and cell line authentication. Because of the low number of examples, both entity types had highly variable F1 scores that were lower than we would have liked. In the future, we plan to create an expanded dataset to improve these numbers. However, the simple fact that our curators struggled finding these types of statements in the literature shows that these key rigor criteria are severely underreported.

Overall, 11 curators have worked on annotation over the last 4 years ranging from early- to late-career researchers. When initially developing SciScore classifiers, an inter-curator agreement (ICA) was calculated between J.M. and A.B. to determine the feasibility and difficulty level of the curation tasks (>90%). In cases where there was not complete agreement, curators would discuss until an agreement was reached forming the basis for our initial curation rules. All new curators were expected to annotate with more than 90% agreement with J.M. For each subsequent training file, J.M. (with A.B. advising) would serve as both quality control and as a point of contact for other curators to ensure a high ICA was maintained over the course of the training.

Antibodies are composite entities that can use detected antibody metadata to improve recognition of the antibody entity. The antibody composite entity identification relies on the presence of some of these features in a short span of text (within 3 word-phrases). For example, in Figure S1, the various antibody components are visualized mimicking SciScore's training and entity detection. When an antibody name cannot be found in a sentence, the presence of an antibody RRID will trigger a second pass with a reduced threshold for detection as SciScore now has reason to suspect an antibody is described within the sentence. In cases where no RRID is mentioned, SciScore attempts to use the detected name and metadata to suggest an RRID when possible. We assume that the authors will report some but not all antibody features for any given antibody. Treating the antibody name feature as its primary tag, the overall F1 score for antibodies is 78.9 with a precision of 86.9 and a recall of 72.5.

While the cell line algorithm has been tested previously to find the total number of cell lines used throughout the open access subset of PubMed Central (Babic et al., 2019) and the software/database detector has been previously described in detail (as well as its features and data representation) (Ozyurt et al., 2016), the other algorithms had not been thoroughly validated before this on complete articles outside of the training set. To validate SciScore's total performance, we tested SciScore against an independent set of human-curated data. This set was created using 250 papers randomly chosen using the random() function in SQLite (RRID:SCR_017672) from our dataset of open-access papers. We did not perform a power analysis to determine if this number was sufficient, but chose a round number that was larger than any of our power calculations for individual journals. Each paper was manually reviewed by a curator (N.A., an early-career researcher, with oversight from J.M and A.B.) to determine which rigor criteria and key resource information had been referenced. For each paper, the methods section was read, and the curator noted the presence or absence of each entity type listed in Table S1. For this check, the curator and SciScore were considered to be in agreement if both had marked an entity type as either present or absent. We note that this criteria is substantially less stringent than what we used to assess F1 rates (shown in Table S1), where the exact entity had to be detected by the tool with 0 edit distance to consider the match a "hit". We assumed that if both the curator and SciScore agreed about the presence or absence of an entity type, then the answer was correct and we did not look more deeply into these data. If there was a disagreement, it would then be classified as either a false negative error or a false positive error with the assumption that the curator is always correct. False negatives were defined as cases where the classifier incorrectly noted an entity type as absent when it was in fact present. False positives were defined as cases where the classifier noted an entity type as present when it was missing. For example, if a paper containing multiple antibodies was noted by the curator as having antibodies present and SciScore determined that there were antibodies present as well, then this would be considered an agreement. In that example, if SciScore had determined that no antibodies were present, then this would be considered a false negative error. Note that the curator did not keep track of exactly which antibodies were used in the paper or how many. For this analysis, the curator was blinded to the output of the algorithm while curating papers in this set. For validation, this information was then compared against our calculated SciScore classifier performances, listed in Table S1; the results of this analysis are in Table S2.

Scoring Framework

All research papers in the OA subset were scored on a 10-point scale. To calculate the total score for each paper, the scoring was broken down into two equally weighted sections: 5 points for rigor adherence (made up of the rigor

criteria listed in Table 1) and 5 points for key resource identification (consisting of the key biological resource types listed in Table 1). In cases where no rigor criteria or key resources were detected, the paper was considered "not applicable" and received a score of 0. Papers given a 0 were excluded from the dataset because in cases where the algorithms cannot find any criteria to judge, there is no way of determining if a score is appropriate. As SciScore was originally intended for biomedical research articles, papers scored as 0 typically fall far outside of its current scope (e.g. X-ray crystallography), or are the wrong paper type (e.g. a letter to the editor). Indeed, of the 197,892 not applicable (0 scoring) papers, over 30,000 came from the following five journals: Acta crystallographica. Section E, Structure reports online (98% of articles scoring 0), Nanoscale research letters (71%), Beilstein journal of organic chemistry (78%), Acta crystallographica. Section E, Crystallographic communications (95%), and iScience (100%) (Data S6). In order to validate this assumption, a second set of human-curated data was created using 250 papers that had received a score of 0. These papers were randomly chosen using the random() function in SQLite. Each paper was then manually reviewed by a curator (J.M. with oversight from A.B.) to determine if any rigor criteria had been mentioned and which key resources, if any, had been referenced. Similar to our scored paper analysis, any criteria found was marked as either present or absent. The curator was not blinded to the output of the algorithm for this set, which may introduce an element of bias for this portion of the analysis.

Of the 250 "not applicable" papers, 81.2% were found to have been correctly scored (n = 203). Of these 203 papers, 5 were found to be using supplementary methods sections, so a human might be able to look at these, but these sections are invisible to our algorithm, so we did not consider these a miss; 6 had their experimental procedures broken up across different sections of their papers, while 6 did not contain a clear methods sections at all. 47, or 18.8%, of the "not applicable" papers were found to have been incorrectly scored, that is, they were within scope, but the algorithm did not detect any relevant entity. Of these 47 incorrectly scored papers, 45 were found to contain at least one software tool that was not detected by SciScore. This was by far the most missed entity in this set of papers. Blinding and sex as a biological variable were each missed by SciScore in 3 papers, while IRB/Consent, IACUC, blinding, and organism entity types were each found to only have been missed in one paper. These values all fall in line with what was expected based on our calculated rates for false negatives (shown through the recall rate in Table S1). The relatively low agreement rate for software tools seems reasonable as new software tools are often created with a specific use in mind and, as a result, are sometimes only used a handful of times. Because of this, there is a high number of uncommon software tools with which SciScore has very little tool specific training data. This leads to a higher rate of false negatives for those types of software. However, this issue only impacts uncommonly used or recently created software. As a result of these analyses, we did not seek to tune parameters further for SciScore.

We note that when creating the manually checked datasets, we grouped IRB and consent as well as cell line authentication and contamination statements so the coding would be consistent with the output of the automated pipeline. This means that we counted the presence of one of these entity types as sufficient for both. Of these entity types though, all can be considered conditional and are therefore not entirely independent; e.g., studies that require IRB approval usually require a statement of consent; studies using cell lines normally require both an authentication statement and a contamination statement. Because of this, we feel that it is not unreasonable to group these criteria together in these instances.

Again, SciScore scores papers using a 10-point scale broken into two equally weighted sections: rigor adherence (5 total points) and key resource identification (5 total points). In general the rigor section score increases for each criterion that is detected. In certain cases, a particular criterion may be deemed irrelevant and is not expected (or scored), such as the cell line authentication statement, which would not be required in papers that do not use cell lines. Currently, we weigh ethical approval sentences (which could be of the following types: IRB, IACUC, or consent statement) as two criteria even if only 1 criteria (i.e. IACUC approval) is found because this tool is intended for manuscripts in preparation and not having a statement on ethics can have serious legal ramifications. In short, simply comparing the total number of found, relevant criteria to the total number of expected, relevant criteria, one

could roughly calculate the score for the rigor section. In short, the rigor section score can be estimated by using the following formula:

$$\frac{detected,\ relevant\ criteria}{expected,\ relevant\ criteria} \times 5 \qquad \text{(Eq. 1)}$$

This presents a positive bias in scores towards vertebrate animal and human subjects papers that include the ethical approval statement, and a negative bias against cell line and invertebrate papers, as ethical approval is not required in those cases. The current version of the tool does not score cell line authentication if no cell line is detected, but does not yet have the ability to distinguish whether ethical approval is necessary.

The key resources section is scored altogether as one block and takes into consideration the total number of resources found using a similar found:expected ratio scoring system. In brief, all detected resources are categorized into two scoring groups: detected but not uniquely identifiable (no points), and uniquely identifiable (full points). We define a resource as "uniquely identifiable" if the entity can be linked to a single resource based on the metadata provided. For example in the sentence "We used the mouse monoclonal GFAP antibody from Sigma", the algorithm is likely to detect a single antibody and vendor, but the catalog number or research resource identifier (RRID) would not be found. For this sentence, this resource would not contribute any points towards the "found" total because the resource is not uniquely identifiable. It would, however, still contribute towards the expected resources count, so if this was the only resource detected, the author would receive a 0 of 5 for this section. If the author were to provide a catalog number, the algorithm may suggest a RRID given that it is able to estimate with a high level of confidence a single RRID entity with matching metadata (suggestions are granted points for the identifiable section), a valid matching RRID also guarantees the point. We then calculate the key resource section's score using a similar formula as the rigor section where the numerator is the number of identifiable resources and the denominator is the total number of resources detected. When the algorithm fails to recognize a resource, that is considered a false negative, occuring at rates outlined in Table S1. We note that the values reported in Table S1 are for individual entities. When an entity is discussed several times, the probability should be additive. Papers tend to discuss resources several times in the methods section; for cell lines, each cell line was mentioned twice on average, improving the rate of resource identification in the paper. Because of this, we expect that our SciScore to curator agreement scores should be at or above the raw values. Final scores are rounded to the nearest integer.

Impact Factor Comparison

All journals contained in the OA subset were initially considered for our analysis. In order to ensure that the average score calculated for each journal was representative of their true average, we limited our analysis to journals with sample sizes larger than the minimum required sample size calculated for each journal. Journals that did not meet this minimum were excluded from our analysis. We then searched the Journal Citation Reports (JCR, RRID:SCR_017656) database (operated by Clarivate Analytics; RRID:SCR_017657) to obtain the journal impact factor (JIF) and average JIF percentile for each journal's 2018 scores. These values are the most recent obtainable scores as new JIF information is usually released ~6 months after the end of the year (e.g. JIF values for 2019 will be released around June of 2020). Searches were performed in November of 2019. Journals that did not have their information listed in the JCR were excluded from our analysis. JIF is "calculated by dividing the number of current year citations to the source items published in that journal during the previous two years" according to Clarivate Analytics, the official source for JIFs. For JIFs in 2018, this roughly translates to the following equation (Eq. 2):

$$\frac{Citations_{Articles\ 2017} + Citations_{Articles\ 2016}}{Articles_{2017} + Articles_{2016}} \qquad \text{(Eq. 2)}$$

Because of this, when we calculated the average score for each journal, we only included scores from 2016 and 2017, so that the SciScores and JIFs would theoretically be representative of roughly the same papers. We say "roughly" because JIF is calculated using "citable items", a vague term sometimes made up of a variety of article

types (original research, commentaries, opinions, etc.),[43] while SciScore is currently intended for use on original research only. The average JIF percentile is calculated using the rank of each journal's impact factor grouped by the field in which the journal is indexed. This calculation accounts for citation variations between different scientific fields as the JIF percentile only compares journals within a specific category (cell biology journal vs. cell biology journal). As a result, any difference in citation counts between fields (e.g. physical chemistry vs. immunology) will be mitigated, allowing for a better comparison across all biomedical research. SciScore percentile was calculated based on the average SciScore of all 490 journals used in our impact factor comparison. In order to evaluate the correlations between JIF vs. SciScore and JIF percentile vs. SciScore percentile, we used Sheets (Google Sheets; RRID:SCR_017679) to calculate Spearman's rank-order correlation for each. Spearman's correlation was chosen over Pearson's because we did not assume bivariate normality. Some potential sources of biases affecting this analysis are the FUTON (full text on the Net) bias and the NAA (no abstract available) bias, which in both cases can positively impact citation counts for open-access research, while negatively impacting the number of citations for research not freely available on the web (Mueller et al., 2006; Murali et al., 2004; Wentz, 2002). We feel though that any impact associated with these biases would be mitigated because a vast majority of the journals analyzed here were at least partially open-access and all cases where abstracts were not available were universally excluded.

Statistics

To determine if a journal sampling was representative of its population in our impact factor analysis, we calculated the minimum sample sizes (n) required for each journal using the following equation (Eq. 3) for the sample size estimation of a finite population:

$$n = \frac{\frac{z^2 \cdot \widehat{p}(1-\widehat{p})}{\varepsilon^2}}{1 + \frac{z^2 \cdot \widehat{p}(1-\widehat{p})}{\varepsilon^2 \cdot N}} \quad \text{(Eq. 3)}$$

where z is the z score, $\widehat{p}$ is the sample proportion, ε is the confidence interval, and N is the population. We used a confidence level of 95%, a confidence interval of 5%, and a sample proportion of ~0.875, which was the proportion of papers in our dataset that received a score above 0. Population sizes varied, but were determined by performing searches on PubMed restricted by publication type [journal article] and journal name. The minimum sample size was also calculated for each year to determine how far back our analysis should consider. For those calculations, the population was determined by the number of journal articles published in PubMed for a given year. These calculations were performed in Sheets. For each manually curated test set, a set size of 250 was chosen arbitrarily as a round number that was larger than the minimum sample size calculated using equation 3. For all other analyses, journals were only included if more than 10 papers were scored per year unless stated otherwise. For the antibody identification analysis, we only included journals that had more than 10 scored papers containing at least one antibody in a given year.

For SciScore named-entity classifiers, we used the standard measures used to quantify performance: recall (R), precision (P), and the harmonic mean of R and P (F1). These were determined by the following formulae:

$$R = \frac{True\ Positives}{True\ Positives + False\ Negatives} \quad \text{(Eq. 4)}$$

$$P = \frac{True\ Positives}{True\ Positives + False\ Positives} \quad \text{(Eq. 5)}$$

$$F1 = \frac{(2 \cdot P \cdot R)}{(P + R)} \quad \text{(Eq. 6)}$$

In this case, false negatives are criteria that were missed by SciScore but were labeled by a human curator, and false positives were incorrectly identified text labeled by SciScore.

We did not obtain an institutional review board approval to conduct this study as we did not utilize any human or animal subjects, making this study exempt.

Supplemental References

Babic, Z., Capes-Davis, A., Martone, M., Bairoch, A., Ozyurt, B., Gillespie, T., Bandrowski, A. (2019). Incidences of problematic cell lines are lower in papers that use RRIDs to identify cell lines. eLife 8, e41676.

Mueller, P., Murali, N., Cha, S., Erwin, P., and Ghosh, A. (2006). The effect of online status on the impact factors of general internal medicine journals. Neth. J. Med. 64, 39-44.

Murali, N., Murali, H., Auethavekiat, P., Erwin, P., Mandrekar, J., Manek, N., Ghosh, A. (2004). Impact of FUTON and NAA Bias on Visibility of Research. Mayo Clin. Proc. 79, 1001-1006.

Ozyurt, I., Grethe, J., Martone, M. and Bandrowski, A. (2016). Resource Disambiguator for the Web: Extracting Biomedical Resources and Their Citations from the Scientific Literature. PLoS One 11, e0146300.

Wentz, R. (2002). Visibility of research: FUTON bias. Lancet 360, 1256.
